# Interstitial Nephritis: A Change in Diagnosis With Next-Generation Sequencing

**DOI:** 10.1016/j.ekir.2022.01.1061

**Published:** 2022-02-02

**Authors:** Mira Choi, Anne Rübsam, Marten Schulz, Eva Decker, Anja Friedrich, Eva Schrezenmeier, Fabian Halleck, Kai-Uwe Eckardt, Carsten Bergmann

**Affiliations:** 1 Department of Nephrology and Medical Intensive Care, Charité-Universitätsmedizin Berlin, Corporate Member of Freie Universität Berlin and Humboldt-Universität zu Berlin, Berlin, Germany; 2 Department of Ophthalmology, Charité-Universitätsmedizin Berlin, Corporate Member of Freie Universität Berlin and Humboldt-Universität zu Berlin, Berlin, Germany; 3 Berlin Institute of Health (BIH), Berlin, Germany; 4 Department of Hepatology and Gastroenterology, Charité-Universitätsmedizin Berlin, Corporate Member of Freie Universität Berlin and Humboldt-Universität zu Berlin, Berlin, Germany; 5 Medizinische Genetik Mainz, Limbach Genetics, Mainz, Germany; 6 Department of Medicine, Nephrology, University Hospital Freiburg, Freiburg, Germany

## Introduction

Kidney transplantation remains the best treatment option in patients with chronic kidney failure. However, the underlying disease leading to kidney failure considerably influences short- and long-term renal transplant outcome and poses a potential risk for disease recurrence within the transplant. We still face a significant proportion of patients with undiagnosed kidney disease, ranging from 20% to 40% in patients with chronic renal insufficiency or patients with kidney failure on the waiting list.^1^, ^2^, ^3^ The advent and spreading of new sequencing technologies known as next-generation sequencing or massively parallel sequencing have significantly improved diagnostic strategies for inherited kidney diseases. While the clinical and genetic heterogeneity of many hereditary nephropathies poses a major challenge, the identification of the exact genetic cause of a disease is of crucial importance for therapy, care, and prognosis (as summarized in Table 1). The hope of genomics-driven medicine is to provide increasingly personalized treatment options based on a person’s exact genetic information. Implementing genetics early in the diagnostic algorithm may substantially reduce the diagnostic odyssey. We report on a male patient who had undergone a kidney transplant and was clinically and histologically diagnosed with interstitial nephritis at the age of 13, in whom we identified compound-heterozygous pathogenic variants in the ciliopathy-related gene *NPHP3* almost 30 years later.

**Table 1:** Potential implications of genetic findings in patients with CKD

Identification of pathogenic genetic variants in CKD-associated genes may influence medical management of patients, including prognostic information, recurrence risk, and clinical workup for extrarenal manifestations and has the potential for further therapeutic implications.
Genetic findings might lead to further screening of close relatives, with the opportunity for further medical advice and to allow early diagnosis and by that to avoid complications.
Genetic findings might have an impact for family planning.
The identification of pathogenic CKD gene variants can provide diagnostic information complementary to, or instead of, a kidney biopsy.
In case of kidney transplantation, the risk of disease recurrence can be taken into account, enabling early and specific intervention.
Kidney donation from living related donors to family members with a dominant pathogenic variant should be avoided.

CKD, chronic kidney disease.

**Table 2:** Teaching points

A significant proportion of patients (20%–40%) with chronic renal insufficiency or kidney failure remains with undiagnosed kidney disease.
New sequencing technologies known as NGS or MPS significantly improved diagnostic strategies for inherited kidney diseases.
The identification of the exact genetic cause of a disease is of crucial importance for therapy, care, and prognosis.
Pathogenic variants in *NPHP* genes may present as isolated kidney disease, but pleiotropic manifestations with a broader phenotypic multisystem disease should be kept in mind.

MPS, massively parallel sequencing; NGS, next-generation sequencing.

## Case Presentation

A 42-year-old male diagnosed with kidney failure at the age of 13 without any previous symptoms except for a slight icterus received a deceased donor kidney transplantation shortly after, at the age of 14. Kidney biopsy performed at that time revealed interstitial nephritis. Renal transplant function remained stable throughout follow-up, with a baseline creatinine of 1.3 mg/dl. Family history was negative for kidney diseases, and he was an only child of healthy parents. The patient himself had 2 children aged 2 and 5 years, for which the question of any recurrence risk had been raised by him and his wife.

We decided to perform next-generation sequencing-based genetic diagnostic testing to elucidate the etiology of his kidney disease and identified compound heterozygosity for *NPHP3*. On his paternal allele, the propositus carries the heterozygous silent *NPHP3* variant c.2154C>T that does not directly lead to an amino acid change [p.(Phe718=)] but has been previously described in several patients with suspected nephronophthisis and chronic kidney failure, each being compound-heterozygous with a pathogenic *NPHP3* variant *in trans* (i.e., on the other parental allele/gene copy) (own unpublished data and Molinari *et al.*^4^). In this study, analysis of renal cell-derived mRNA harboring this alteration demonstrated that the altered allele leads to a loss (skipping) of exon 15 and thus presumably to a frameshift and the emergence of a new stop codon.^4^ This results in either premature degradation of the mRNA (nonsense-mediated decay) or truncation of the protein. In line with its presumed pathogenicity, this alteration has been detected in only 0.001% of the general population (in 282860 alleles only 3× heterozygous; gnomAD). In summary, the above variant can be classified as pathogenic. On the patient’s maternal allele, we detected the heterozygous *NPHP3* 2-base pair deletion c.2702_2703del resulting in a reading frameshift [p.(Phe901Cysfs∗2)]. This results in the emergence of a new stop codon and thus either premature degradation of the mRNA (nonsense-mediated decay) or truncation of the protein. To our knowledge, this variant has not been previously reported in the literature (HGMD 2020.3). Furthermore, it has been detected in only 0.0004% of the general population (in 251390 alleles 1× heterozygous; gnomAD). Overall, this alteration is to be considered pathogenic, too. In conclusion, we could confirm *NPHP3*-related disease in our patient.

We performed extensive workup for NPHP-3 related extrarenal manifestations. The patient denied visual impairments such as night blindness (as seen with retinal degeneration in Senior-Løken syndrome) or any neurologic symptoms (as seen in Joubert syndrome). Subclinical eye involvement could be excluded by thorough ophthalmologic examinations (see Supplementary Methods) as depicted in the Supplementary Figure S1A to C. Slit lamp examination and fundoscopy revealed a regular anterior and posterior segment. Optical coherence tomography revealed no signs of optic disc atrophy or macular degeneration. Furthermore, no signs of early retinal degeneration were detected. Liver ultrasound revealed normal findings and using 2-dimensional shear wave elastography, we did not find increased liver stiffness as an indirect sign of liver fibrosis (Supplementary Figure S1D–E). Cerebral magnetic resonance images and heart ultrasounds were unremarkable, too.

## Discussion

*NPHP3* belongs to a group of currently >20 genes known for nephronophthisis (NPHP), a progressive tubulointerstitial renal disease characterized by reduced urine concentrating capacity, growth retardation, polyuria/polydipsia, and finally, chronic kidney failure that usually develops before adulthood. NPHP and related disorders referred to as ciliopathies are linked to the dysfunction of the primary cilium, a sensory antenna present on most vertebrate cells. Approximately 10% to 20% of individuals with pathogenic variants in *NPHP* genes may show pleiotropic manifestations with a broader phenotypic multisystem disease spectrum than mere isolated kidney disease (e.g., cerebellar hypoplasia and ataxia, skeletal disorders, ophthalmologic abnormalities, in particular, retinitis pigmentosa, liver fibrosis).^5^ However, clinical workup revealed solely renal involvement in our patient.

Our study highlights the increasing importance of sequencing for the discovery of genetic disorders among adult patients (Table 2). Regarding a high prevalence of rare genetic disorders in kidney transplant recipients with an unknown etiology of kidney disease, close interdisciplinary collaboration is of major importance and improved our understanding in the demonstrated case. Recent evidence supports the hypothesis that “phenocopies” could account for a non-negligible fraction of patients who are currently classified as nongenetic, paving the way for a more comprehensive understanding of the genetic background of disease. Although still not inexpensive, a genetic test usually comprises a once-in-a-lifetime performance, which should be considered in cases of uncertainty of the underlying disease. In view of the progress made in this field, we would like to emphasize the aim to clarify any unknown disease in patients with kidney failure as knowledge might have potential implications for treatments before and after transplantation.

We utilized a targeted comprehensive panel approach in our study, which has pros and cons compared with an exome-wide approach recently discussed in greater detail elsewhere.^6^ Overall, we put major efforts in our next-generation sequencing methodology to find a compromise and established a customized gene panel that targets all 600 genes described and associated with kidney disease or allied disorders, as well as corresponding flanking intronic sequences. The panel design is constantly updated by surveillance of current literature as well as enriched by targets in noncoding regions for described variants listed in well-accepted databases such as HGMD or ClinVar.

Results such as those obtained in the current case have several implications for our patient. In contrast to patients with immune-mediated diseases, the prognosis in individuals with most genetic kidney disorders is good, with a lack of recurrence. Moreover, therapeutic approaches with steroids and immune suppressive drugs are useless or even contraindicated in patients who harbor a mutation in an onco- or tumor suppressor gene. Likewise, kidney donation by a family member carrying a dominant mutation is contraindicated and needs to be avoided. In contrast, heterozygous carriers for autosomal recessive disorders such as in our case can be accepted as kidney donors. Finally, indirect consequences arise for family counselling. For our patient and his nonrelated wife it came as a huge relief to learn that there is practically no recurrence risk for their 2 healthy children.

## Disclosure

CB holds a part-time faculty appointment at the University of Freiburg in addition to his employment with the Limbach Group for which he heads and manages Limbach Genetics. All other authors declared no competing interests.

## Patient Consent

The authors declare that they have obtained consent from the patient and his parents discussed in the report.

## Supplementary Material

**Supplementary Methods.**

**Figure S1.** Fundus images (A) and optical coherence tomography (OCT) images of the macula (B) and optic disc (C) of the right eye (left panel) and left eye (right panel). Fundus image (B) depicting a regular fundus appearance. The representative OCT scan of the foveal region (B) shows a regular macula. The OCT scan of the optic disc (C) with the near infrared image of the optic disc above and the retinal nerve fiber layer thickness map below reveals a normal optic disc without any signs of optic atrophy. 2D -Shearwave elastography. Color -coded elastogram displayed in real -time (D). Propagation of shear waves depicted by parallel lines (E).

**Supplementary References** (PDF).
