# Supplementary material for: Interstitial Nephritis: A Change in Diagnosis With Next-Generation Sequencing
Source: Kidney Int Rep. 2022 Feb 2;7(5):1128–30. doi: 10.1016/j.ekir.2022.01.1061 (PMC9091579; doi:10.1016/j.ekir.2022.01.1061)
Supplement: Supplementary File (PDF) [file mmc1.pdf]

Supplementary Material

Supplementary Methods

NGS technologies and comprehensive bioinformatic analyses utilized in this project are described in detail elsewhere [1,2]. In brief, we utilized a customized sequence capture library (e.g. by Twist Bioscience©) with curated target regions - currently comprising more than 600 genes described and associated with kidney disease or allied disorders- as well as corresponding flanking intronic sequence according to the manufacturer´s recommendations. The panel design is constantly updated by surveillance of current literature as well as enriched by targets in non-coding regions for described variants listed in well-accepted databases like HGMD or ClinVar. Moreover, the design is optimized in low-performance regions as well as in critical regions like in PKD1 as described [1]. DNA samples were pooled and sequenced in a multiplexing procedure. DNAs were enriched using a sequence capture approach, and sequenced using Illumina sequencing-by-synthesis technology with an average coverage of more than 300X for a targeted panel setup. Raw data were processed according to bioinformatics best practise procedures. Mapping and coverage statistics were generated from the mapping output files using standard bioinformatics tools (e.g. Picard). High and reproducible coverage achieved by our sequencing approach enabled copy number variation (CNV) analysis. Performance of the wet-lab and bioinformatic processes are validated and controlled according to national and international guidelines [3,4] reaching high sensitivity for SNV, Indels and CNVs using well-established reference samples as well as a large cohort of positive controls, especially for CNVs. For interpretation of identified variants, we have developed own published bioinformatic algorithms using a stepwise filtering process conducted by an experienced team of scientists and supported by various bioinformatics decision tools. Sequence variants of interest were verified by Sanger sequencing if NGS results failed internal validation guidelines.

Ophthalmologic assessments included visual acuity testing using a decimal chart, anterior and posterior segment examination, spectral-domain optical coherence tomography of the optic nerve and macula (SD-OCT; Heidelberg Spectralis, Heidelberg Engineering, Heidelberg, Germany), fundus autofluorescence (Clarus 700, Carl Zeiss Meditec AG, Jena, Germany) and ERG recordings (Welch Allyn, Inc., Skaneateles Falls, USA).

Full- field electroretinogram (ERG) was used assess the status of the retina. Pupils were pharmacologically dilated and participants underwent 20 min dark adaptation prior to commencement of stimuli. The stimuli delivered were the standard International Society for Clinical Electrophysiology of Vision (ISCEV) dark-adapted stimuli (flashes delivering 0.01, 3.0 and 10 photopic cd/m2 s, conventionally termed the dark adapted (DA) 0.01, DA 3 and DA 10), followed by additional white flashes (delivering 0.67, 4.0, 13 and 67 photopic cd/m2 s). The signals were collected by a sensor strip skin electrode affixed to the lower eyelid of both eyes. The amplitudes of the a- and b-waves and sums of the oscillatory potential (OP) amplitudes and implicit times from the OP1 to OP5 were measured.

Liver stiffness was indirectly determined by measuring the velocity of induced shear waves since the speed of shear wave propagation is proportional to tissue elasticity. 2D- Shear wave elastography (2D-SWE) measurement was performed as proposed by guidelines using the Canon (former Toshiba) Aplio 500 ultrasound system (Canon Medical systems Corporation, Otawara, Tochigi, Japan) [5]. 2D-SWE value was defined as median value of at least five reliable measurements. Liver stiffness was expressed in meters per second (m/S) or converted into kilopascals (kPa).

Supplementary Figure S1

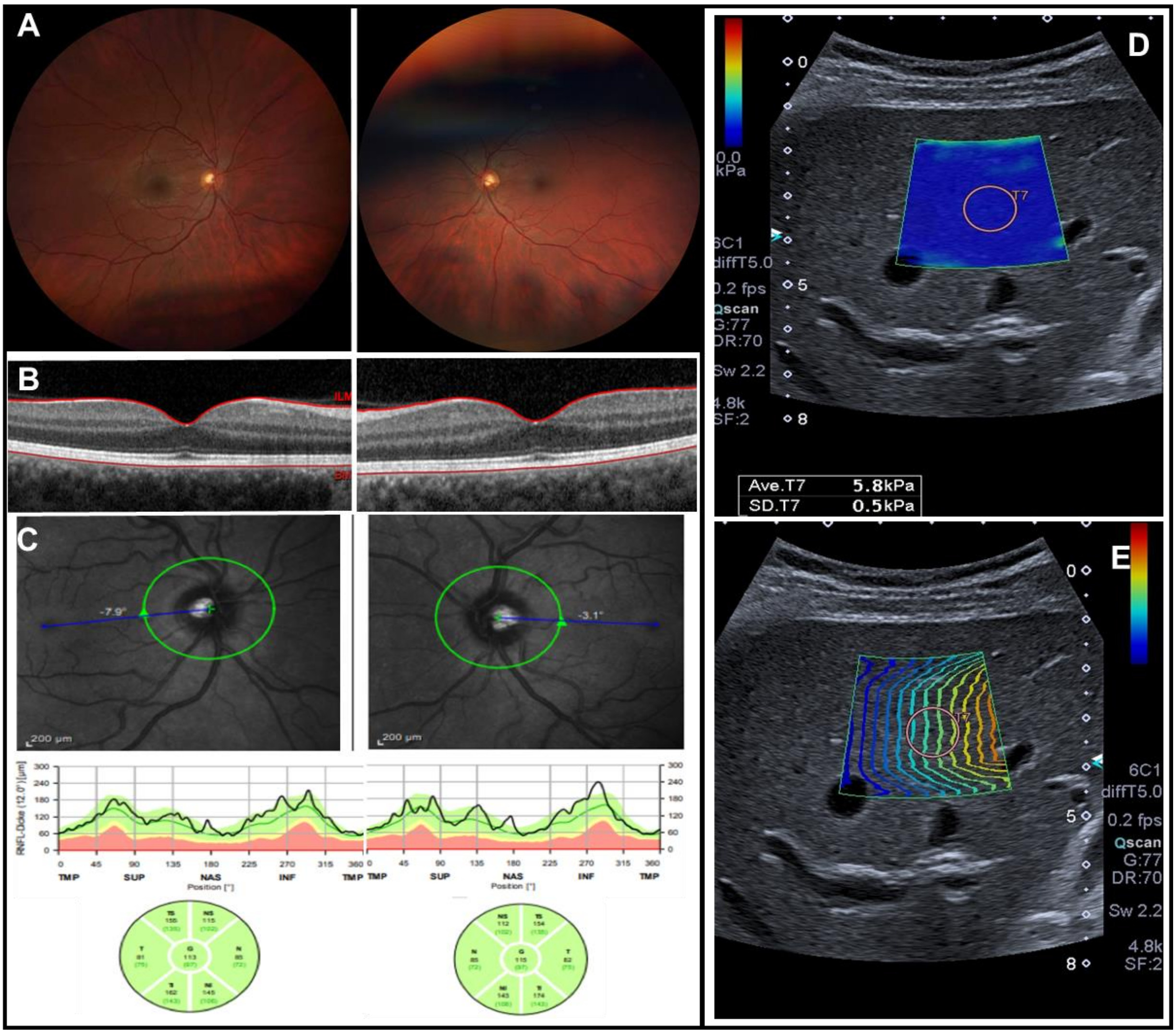

Supplementary Figure legend

Supplementary Figure S1: Fundus images (A) and optical coherence tomography (OCT) images of the macula (B) and optic disc (C) of the right eye (left panel) and left eye (right panel). Fundus image (B) depicting a regular fundus appearance. The representative OCT scan of the foveal region (B) shows a regular macula. The OCT scan of the optic disc (C) with the near infrared image of the optic disc above and the retinal nerve fiber layer thickness map below reveals a normal optic disc without any signs of optic atrophy. **2D-Shearwave elastography.** Color-coded elastogram displayed in real-time **(D).** Propagation of shear waves depicted by parallel lines **(E).**

Supplementary References

1. Eisenberger T, Decker C, Hiersche M, et al. An efficient and comprehensive strategy for genetic diagnostics of polycystic kidney disease. *PLoS One*. 2015;10(2):e0116680. doi:10.1371/journal.pone.0116680
2. Lu H, Galeano MCR, Ott E, et al. Mutations in DZIP1L, which encodes a ciliary-transition-zone protein, cause autosomal recessive polycystic kidney disease. *Nat Genet*. Jul 2017;49(7):1025-1034. doi:10.1038/ng.3871
3. Matthijs G, Souche E, Alders M, et al. Guidelines for diagnostic next-generation sequencing. *Eur J Hum Genet*. Oct 2016;24(10):1515. doi:10.1038/ejhg.2016.63
4. Rehm HL, Bale SJ, Bayrak-Toydemir P, et al. ACMG clinical laboratory standards for next-generation sequencing. *Genet Med*. Sep 2013;15(9):733-47. doi:10.1038/gim.2013.92
5. Dietrich CF, Bamber J, Berzigotti A, et al. EFSUMB Guidelines and Recommendations on the Clinical Use of Liver Ultrasound Elastography, Update 2017 (Long Version). *Ultraschall Med*. Aug 2017;38(4):e16-e47. EFSUMB-Leitlinien und Empfehlungen zur klinischen Anwendung der Leberelastographie, Update 2017 (Langversion). doi:10.1055/s-0043-103952
